# A risk screening tool for ethical appraisal of evidence-generating initiatives

**DOI:** 10.1186/s12910-015-0039-3

**Published:** 2015-07-07

**Authors:** Nancy K. Ondrusek, Donald J. Willison, Vinita Haroun, Jennifer A. H. Bell, Catherine C. Bornbaum

**Affiliations:** Public Health Ontario | Santé publique Ontario, 480 University Avenue, Suite 300, Toronto, ON M5G 1 V2 Canada; Institute of Health Policy, Management and Evaluation, University of Toronto, 155 College Street, Suite 425, Toronto, ON M5T 3 M6 Canada; Department of Clinical Epidemiology & Biostatistics, Faculty of Health Sciences, McMaster University, 1280 Main St. W., Hamilton, ON L8S 4 L8 Canada; Ontario Long Term Care Association, 425 University Avenue, Suite 500, Markham, M5G 1 T6 Canada; University Health Network, Bioethics Programme and Department of Psychosocial Oncology and Palliative Care, Princess Margaret Hospital, 610 University Ave., Toronto, ON M5T 2 M9 Canada; Department of Psychiatry, University of Toronto, 250 College Street, 8th Floor, Toronto, ON M5T 1R8 Canada; Health & Rehabilitation Sciences, University of Western Ontario, Elborn College, Room 1041, London, ON N6A 1H1 Canada

**Keywords:** Risk screening tool, Risk assessment, Risk, Ethics, Research, Quality improvement, Program evaluation, Evidence generating, Learning healthcare, Public Health

## Abstract

**Background:**

The boundaries between health-related research and practice have become blurred as initiatives traditionally considered to be practice (e.g., quality improvement, program evaluation) increasingly use the same methodology as research. Further, the application of different ethical requirements based on this distinction raises concerns because many initiatives commonly labelled as “non-research” are associated with risks to patients, participants, and other stakeholders, yet may not be subject to any ethical oversight. Accordingly, we sought to develop a tool to facilitate the systematic identification of risks to human participants and determination of risk level across a broad range of projects (e.g., clinical research, laboratory-based projects, population-based surveillance, and program evaluation) and health-related contexts. This paper describes the development of the Public Health Ontario (PHO) Risk Screening Tool.

**Method:**

Development of the PHO Risk Screening Tool included: (1) preparation of a draft risk tool (n = 47 items); (2) expert appraisal; (3) internal stakeholder validation; (4) external validation; (5) pilot testing and evalution of the draft tool; and (6) revision after 1 year of testing.

**Results:**

A risk screening tool was generated consisting of 20 items organized into five risk domains: Sensitivity; Participant Selection, Recruitment and Consent; Data/Sample Collection; Identifiability and Privacy Risk; and Commercial Interests. The PHO Risk Screening Tool is an electronic tool, designed to identify potential project-associated risks to participants and communities and to determine what level of ethics review is required, if any. The tool features an easy to use checklist format that generates a risk score (0–3) associated with a suggested level of ethics review once all items have been completed. The final score is based on a threshold approach to ensure that the final score represents the highest level of risk identified in any of the domains of the tool.

**Conclusions:**

The PHO Risk Screening Tool offers a practical solution to the problem of how to maintain accountability and appropriate risk oversight that transcends the boundaries of research and practice. We hope that the PHO Risk Screening Tool will prove useful in minimizing the problems of over and under protection across a wide range of disciplines and jurisdictions.

**Electronic supplementary material:**

The online version of this article (doi:10.1186/s12910-015-0039-3) contains supplementary material, which is available to authorized users.

## Background

Traditionally, the distinction between research and practice has been the factor that determines whether formal ethical scrutiny is applied to human subjects-related inquiries [1]. This long-accepted distinction has shaped the development of research ethics regulations, guidelines, and institutional infrastructure supporting ethics oversight, which have been targeted exclusively to initiatives meeting a definition of “research”. As a result of operational and ethical challenges, however, the appropriateness of this dichotomous approach has increasingly been called into question.

Operationally, implementing procedures for ethics review requires the development of definitions or criteria to separate research from practice. In reality, however, the boundary between these two activities is not clear, and attempts to develop criteria to reliably separate them have proven unsatisfactory [2–4]. The blurring of boundaries between research and practice has worsened in recent years, as initiatives traditionally considered practice, such as quality improvement and program evaluation, increasingly use the same rigorous methodologies as research [5]. Moreover, current trends toward the transition of medical care to a “learning health care system” [6], in which the provision of care is closely integrated with observational and comparative effectiveness research [7] or other pragmatic clinical trials [8], further challenges the research-practice distinction.

Apart from the challenge in identifying criteria to reliably distinguish between research and practice, the application of different ethical requirements based on this distinction raises ethical concerns because many of the initiatives that are commonly labelled as “non-research” are associated with risks to patients, participants, and other stakeholders yet may not be subject to any form of ethical oversight [3, 9–11]. Concern that there has been over protection in some areas and under protection in others has led to calls for the creation of “a new ethical foundation…that provides oversight that, rather than being based on a distinction between research and practice, is commensurate with risk and burden in both realms” [12].

Adoption of a risk-based system of oversight that applies to both research and other type of data collection that may be classified as practice, may address the definitional and ethical challenges noted above, but raises new questions regarding how to operationalize such an approach. Key challenges include determining the level of risk associated with various initiatives, and what type of oversight is appropriate. In addition, successful adoption of a broader scope of ethics review requires emphasis on a culture of integrity, where consideration of the ethical implications is accepted by project teams as integral to the planning and conduct of all evidence-generating initiatives involving human participants, rather than something to be considered only as needed to comply with externally dictated regulatory requirements. The term “evidence-generating initiative” is used here to refer to all projects or activities that produce information through systematic collection or use of data about people, their environments, and the health or social services that they receive or provide.

A number of tools have been developed to assess research-related risks, but there are limitations in their ability to support a broader scope of ethics review and to promote a culture of ethical integrity. Risk matrices are used by many research institutions to appraise risk and determine review level (e.g., University of Toronto [13]), but these are designed to sort projects into one of two ethics review streams that have been developed specifically for research (i.e., delegated or full board ethics review). A simple shunting of all projects into existing research ethics review mechanisms would not be appropriate as it would not address concerns about over protection of very low risk projects, and thus would create an unnecessary burden for investigators and ethics oversight systems. Over protection can also interfere with delivery of care, thereby creating rather than reducing risk. [14]. In addition, matrices require that users assign a risk level (e.g., low, medium or high) to the project overall, but do not aid in this assignment through the identification and rating of risks.

Various screening tools are also in use, which sort projects based on the presence or absence of procedures that may be associated with risk, such as the testing of new medical treatments or the use of questionnaires probing sensitive issues (e.g., ARECCI [15]; King’s College London [16]; Vanier College [17]). Although these tools provide more guidance in the assessment of risk, currently available versions involve sorting of projects into “research” or “non-research” designations to determine the appropriate level of ethics oversight. In addition, the tools tend to allow for only a “yes” or “no” response regarding various initiatives, which does not allow for the incorporation of context into determining a project’s risk level. For example, collection of direct identifiers from participants raises privacy concerns that typically warrant additional scrutiny. However, the level of concern when identifiers are collected only for the purose of obtaining and documenting consent, is less than if findings will be associated with identifiable individuals. A simple “yes” to collection of direct identifiers could lead to an overestimation of risk in the first instance, or an underestimation in the second scenario. The ability to consider context improves the specificity of risk assessment, which is valuable in any ethics oversight system, but which would be particularly important in supporting ethics oversight across the research-practice continuum.

Finally, current screening tools reflect the clinical trials origins of research ethics guidelines, and focus on risks mainly from the perspective of individual participants. These tools are not as sensitive in considering risks to collectivities that may arise with health-related evidence generation, particularly in areas such as health services research, public health, implementation science and quality improvement initiatives.

To promote routine incorporation of ethical reflection across health-related settings, we aimed to create a tool that supports identification of risks and determination of risk level that would be efficient, user friendly, and applicable to a broad range of evidence-generating initiatives (e.g., laboratory-based projects, population-based surveillance, program evaluation), including research and other practices. In line with these objectives, this paper describes the development of the Public Health Ontario (PHO) Risk Screening Tool.

## Method

### Setting

PHO is an arm’s length government agency charged with the protection and promotion of health and the reduction of health inequities for Ontarians [18]. This mandate is achieved through the provision of scientific and technical expertise to clients working across health and non-health sectors (e.g., government, local public health agencies, primary care, and professional associations). For example, PHO supports the development of knowledge syntheses, tools and best practice guidelines. In addition, because public health spans a broad range of content areas (e.g., infectious disease, chronic disease, and environmental health) and forms of inquiry (e.g., basic science, clinical trials, health services evaluation, epidemiology, and population health assessment and surveillance), the services offered by PHO are broad in nature.

As evidence generation is frequently a component of routine services as well as research initiatives in this setting, the operational and ethical challenges associated with differential ethics review for research and practice arise frequently. As a result, PHO has developed an integrated system of ethics support and oversight for all evidence-generating health-related initiatives involving human participants or their personal information and/or biological materials [19]. The single, integrated system avoids the challenge of distinguishing between initiatives of research or practice and employs a proportionate risk approach, whereby the level of ethical scrutiny applied to a project reflects the level of risk presented by the project. The system balances institutional responsibility for ethics oversight (e.g., by maintaining compliance with national standards), with the provision of services that promote consideration of ethical impacts throughout the life cycle of a project. Importantly, this proportionate risk approach to ethical appraisal is initiated by use of the PHO Risk Screening Tool, which supports assignment of projects to appropriate review levels.

### Overview of development process

The PHO Risk Screening Tool was designed to promote the systematic consideration of risks for all evidence-generating initiatives involving human participants, their biological material or their data, regardless of whether or not they are classified as “research”, and to support a proportionate approach to ethics review based on risk rather than project type. Development of the PHO Risk Screening Tool included a multifaceted strategy leveraging on the expertise of diverse stakeholders and experts. Specifically, the development process included: (1) preparation of a draft risk tool; (2) expert appraisal; (3) internal stakeholder validation; (4) external validation; (5) pilot testing and evalution of the draft tool; and (6) revision after one year of testing.

#### Preparation of a draft risk tool

A list of activities that may be associated with risk (e.g., collection of direct identifiers, use of documents not available in the public domain, access to private property) was generated through consideration of various ethics guidelines [20] and regulations (e.g., legal mandates of our organization, provincial privacy legislation), our organizational ethics framework [21], existing risk tools [9, 22], and consultation with internal PHO stakeholders. Consultations with internal stakeholders involved meetings with staff including scientists, epidemiologists, and physicians from multiple program areas to elicit feedback about the types of risks that arise in the course of their research and practice. This information was used to inform the nature and scope of potential risks that occur across varied health-related contexts. Based on this review and consultation process, a draft tool was developed. The draft tool included a hierarchy of 13 primary questions to assess potential risks and burdens to participants (e.g., does this activity involve indirect collection or use of personal information?), with 23 secondary questions to clarify the parameters of the potential risk or burden (e.g., will this information be used to contact identified individuals?), and 11 tertiary questions to obtain specific, detailed information about the potential risk or burden (e.g., who will make the first contact with identified individuals?).

#### Expert appraisal

A panel of three reviewers with expertise in research ethics, clinical medicine, and/or public health were asked to individually apply the draft tool to three hypothetical project scenarios that covered a range of content areas, methodological approaches and participant populations. The panel members then came together to discuss their responses with us and comment on the validity, comprehensiveness, and clarity of the measure, and to suggest scores for each item corresponding to the perceived degree of associated risk. Panel members assigned scores of 0, 1, 2 or 3 to each fixed response, to correspond with different levels of ethics review (see Table 1). Differences in opinion were resolved through discussion and consensus. The expert review panel identified potential gaps in comprehensiveness and areas for revision to improve conceptual clarity.

**Table 1:** Summary of attributable risk and review levels for each PHO risk screening tool score

Score	Description of risk Level	Review level
0	No risks identified	Archive
		RST catalogued by ethics office, projects receive periodic audit.
1	Activity appears to be very low risk. Alternatives to ethics board review may be appropriate.	Level 1 Delegated Review
		Delegated review by single reviewer; no completion of separate application form.
2	Activity appears to be minimal risk	Delegated ethics review
		Completion of full ethics review board application form required.
		Review completed by two or more ethics review board members.
3	Activity appears to be greater than minimal risk	Full board ethics review
		Completion of full ethics review board application form required.
		Review completed by full ethics review board.

In keeping with our goal to create a user-friendly tool, it was important that the final product be as short as possible without sacrificing comprehensiveness. To achieve this objective, we analyzed the items to identify generic types of risk that were applicable across diverse methodological approaches and program areas in order to consolidate items if they related to the same generic risk. Using this approach we developed a taxonomy of risks comprising five broad risk domains: Sensitivity; Participant Selection; Recruitment and Consent; Data/Sample Collection; Identifiability and Privacy Risk; and Commercial Interests. The original list of items was checked against the revised tool, to confirm that all previously identified issues were covered and gaps addressed. For operational purposes, an administrative domain was also included, to accommodate requirements for ethics review, such as those imposed by many funding agencies that may apply regardless of risk level. Through this process, a revised, 19-item tool with 5 secondary questions was developed.

#### Internal stakeholder validation

The revised tool was subject to several rounds of testing and revisions with PHO staff to evaluate the Risk Screening Tool and comment on its comprehensiveness, user-friendliness, clarity, and scope. In the first round of reviews the tool was tested by three research operations staff members using the three hypothetical scenarios used for the expert panel discussion, and two staff scientists, using their own projects. The feedback from the five reviewers was used to revise the tool. In the next round, the tool was tested on ten different PHO projects from across program areas (e.g., infectious diseases, health promotion, environmental health and public health laboratories) by two research administrators who each appraised five projects. The tool was revised and then reviewed by an additional three PHO staff members with experience in epidemiology, evaluation, infection control and project management. At this point, the process of internal review and revision was concluded as no new risks were being identified (construct saturation), items had been explicated clearly, and we had reached consensus regarding the assignment of scores for each item.

#### External validation

Following internal appraisal at PHO, representatives from four local public health agencies across the province of Ontario, Canada were also invited to test and review the tool. Reviewer recommendations were synthesized and used to further refine the tool. Similar to the internal appraisal process, assessments with external stakeholders continued iteratively until we were satisfied that all items on the tool were clearly articulated and that the scores for each item were appropriate.

#### Pilot testing of the draft tool

The final version of the tool was pilot tested with a diverse range of projects (e.g., research, surveillance, program evaluation) as part of the launch of PHO’s new integrated ethics review system [19]. In the first year of use, 55 completed Risk Screening Tools were submitted. The administrative burden for implementing the tool was found to be minimal, as a result of the user-completion and automatic scoring features of the tool.

A survey was distributed to PHO scientific and program staff to evaluate the tool, and 22 staff with experience using the tool responded. Time to complete the tool ranged form five to 75 min, with 73 % of respondents taking 20 min or less. Approximately half (47 %) of applicants completed the tool as part of a team of three or more, while 33 % completed the tool on their own. Ninety-one percent of respondents agreed or strongly agreed that the tool was “helpful” and “easy to understand”, although suggestions were offered for clarification of specific items. Respondents were less likely to find the tool “easy to answer” (74 %); comments regarding this question indicated that the tool is appropriate at prompting reflection about risk, but in some cases presented challenges in aligning some items and response options with certain projects.

The results of the user survey, as well as observations by the ethics office regarding items that were associated with misinterpretations or questions from applicants, were used to inform a refinement of the tool. As with the first draft, the revised tool was tested by ethics staff and users and further refined after each round of feedback.

## Results

The resultant PHO Risk Screening Tool (RST 2.0) is an electronic tool, comprising 20 fixed-response items designed to identify potential risks associated with a project and determine what level of ethics review is required, if any (see Additional file 1). The tool features an easy to use checklist format and is intended to be completed electronically by anyone on the project team who is familiar with the details of the project. Supplementary information (e.g., definitions, clarifications) is available by holding the computer cursor over highlighted text. Once all 20 items have been completed, the PHO Risk Screening Tool generates a risk score and provides a suggested level of ethics review. The Risk Screening Tool has two main functions; as an educational tool and as a tool to help sort projects according to risk.

The Risk Screening Tool can serve as an educational tool for investigators and reviewers by helping to increase awareness about the risks that might be associated with health-related evidence-generating initiatives. The Risk Screening Tool can be used at any stage in the planning or implementation of a project and can help encourage ethical reflection among investigators and other project team members. Even for those who are very familiar with the types of risks that may arise, the use of a tool can help ensure that risks are considered systematically.

To support the sorting of projects according to risk level, each of the fixed responses has been assigned a risk level ranging from zero to three. The final score is based on a threshold rather than a cumulative approach. Use of a risk threshold ensures that the final score generated by the PHO Risk Screening Tool will be representative of the highest level of risk identified in any of the five domains of the tool. For instance, if at least one of the responses is scored as “1”, and none of the other responses receive a higher score of “2” or “3”, then the final risk score generated by the tool will be “1”. However, if one or more of the responses corresponded to a risk level of “2”, then the final score would be “2”. The same threshold procedure applies for scores of “0” and “3” whereby the highest level of risk identified, even for a single item, is automatically applied as the final risk score. To reduce the potential influence of score information on user response, these values are not visible to the user (see Additional file 1 for the values associated with each response). The Risk Screening Tool provides guidance to assess the risk associated with a project. Final determination of risk level involves a “validation” step by the research ethics committee (e.g., research ethics board) Chair or delegate to ensure that the assigned review level is appropriate.

The four risk levels (i.e., “0”, “1”, “2”, “3”) identified by the Risk Screening Tool are assigned to different levels of ethical scrutiny, proportionate to the perceived risk (see Table 1). At PHO, we implemented an ethics review process that aims to streamline the review of projects with very low risks or no identified risks (projects scoring “1” or “0”, respectively, on the tool) while ensuring adequate levels of review for projects that may be associated with increased concern (e.g., projects scoring “2” or “3” on the tool). The review process is also compliant with the existing regulatory requirements, which are based on the traditional research-practice distinction paradigm. Projects receiving a score of “2” or “3” are reviewed by our institutional ethics review board (ERB), in a process that is consistent with the traditional practice for projects identified as research [20].

## Discussion

Implementation of a paradigm shift away from the prevailing distinction between research and non-research endeavors requires practical solutions to ensure appropriate risk management practices and ethical scrutiny are maintained. The PHO Risk Screening Tool was developed to facilitate this process through the systematic consideration of risks and the subsequent assignment of proportionate ethics review. The tool has been validated by both internal and external stakeholders representing broad areas of expertise. To date, experiences with the tool confirm that it is appropriate, efficient, and easy to use. Consequently, we believe that the PHO Risk Screening Tool represents a useful and robust measure for appraising risk associated with evidence-generating initiatives in health-related contexts.

As to be expected with the creation of any novel tool, numerous factors served to challenge and facilitate the development process. First, given the diverse range of project types and methods employed across health-related sectors, it was imperative that the tool be comprehensive enough to capture salient risk-related concerns, but also concise enough to prevent feelings of burden by users. The challenge of striking this balance between brevity and scope was addressed by structuring the tool according to general risk domains rather than specific project types. The iterative development and validation processes involving extensive input from stakeholders, service users, and subject matter experts helped to ensure salience and comprehensiveness.

The PHO Risk Screening Tool was developed and validated in a public health setting, which spans a wide variety of content areas (e.g., infectious diseases, chronic diseases and injuries, environmental and occupational health, emergency preparedness, and health promotion), methods of inquiry (e.g., epidemiological surveillance, public health laboratories, program evaluation, among others), and disciplinary backgrounds (e.g., medicine, nursing, laboratory sciences). The tool was also subject to both internal and external review by a diverse range of stakeholders and subject matter experts. Thus, the diverse setting in which the tool was developed and the broad experiences of the tools’ contributors may enhance the generalizability and applicability of the tool to broader settings.

The Risk Screening Tool is reflective of the widely accepted bioethical principles of respect for persons, beneficence, non-maleficence, and to a lesser extent, justice, and so we expect it to be consistent with the conceptualization of risk in other organizations, provinces and countries. The questions in the tool are consistent with concerns identified, for example, in the US regulations for human subjects research, 45CFR46 [23], and the ethics screening questionnaire developed by Massey University, New Zealand [24], such as protection of vulnerable participants, the importance of informed consent where appropriate, and protection of privacy. Similarly, the concept of proportionate review, where the level of ethical scrutiny depends on the risk to participants, is widely accepted, including by the UK National Health Service [25] and US federal regulations. Finally, the tool is meant only to identify potential risks, and to sort projects into one of four review levels. Institutions are free to assign the action associated with each level, including the type of review, and the standards applied in determining the ethical acceptability of risks identified. We thus believe that the tool could be valid in many jurisdictions outside of Canada.

An important goal for this tool was to capture risks to collectives. As such, we have included items that explicity flag identifiability of groups, and possible harms arising from the association of communities or populations with stigmatizing results. These types of risks can persist despite use of de-identified or anonymized data. Additional consideration of risks to collectives is captured indirectly. For example, where consent is required, the project is flagged for additional review using our framework for public health projects [21], which probes about the need for community engagement. However, we recognize that future versions of the tool could be further enhanced by capturing additional risks, such as to a “group’s structure and function because of engagement in research” [26].

In line with our organization’s conceptual ethics framework [21], the PHO Risk Screening Tool was developed to permit review of all evidence-based initiatives across the research-practice continuum. However, it is worth noting that the tool can also be used within the traditional model of ethics review, as demonstrated by the fact that the Risk Screening Tool and accompanying ethics review processes can be used in conformity with current regulatory requirements that are based on the traditional research-practice distinction [20]. Irrespective of an organization’s guiding ethical framework, the ability to sort and appraise the potential risks of myriad projects using a single, comprehensive tool remains valuable. Once sorted, the projects can be processed according to existing institutional structures.

The four-level sorting system offers a number of unique features to facilitate use of the tool across a broad range of projects and settings. Application of the Risk Screening Tool ensures that all evidence-generating initiatives have undergone some level of ethical scrutiny. Level “0” allows exemption from further ethics review based on a systematic approach that can be documented and evaluated. Level “1” allows for the introduction of a less-intensive level of review as an alternative to conventional ethics board submissions. At PHO level 1 review is completed by one reviewer—either a qualified ethics office staff person or an ERB member from a roster of designated level 1 reviewers—and applicants may submit a simple project description rather than filling out the review board application form. This reduces burden on applications and allows for a turn-around-time of a few days rather than two or more weeks. Addition of a level 1 review option for very low risk projects provides an in-between option, avoiding the all-or-none situation of either no ethics review or formal review committee submission for delegated or full board review.

Ultimately, we sought to create a tool that identifies risks associated with evidence-generating initiatives that could be generalizable to diverse health-related settings (e.g., learning health care organizations) and applied at any stage in the review process. Since the tool is systematically applied to all initiatives, it eliminates the need to determine whether a project fits the definition of “research” and therefore would be subject to research ethics review. Consequently, it also provides investigators with documentation of ethics oversight for all health-related evidence-generating initiatives. Minimally, the PHO Risk Screening Tool promotes the systematic assessment of potential risks. It is our hope that the tool will facilitate consideration of ethical concerns early on and by shifting the focus to risk rather than project type, will foster a culture of ethical integrity rather than one of merely compliance.

### Directions for future research

As with the development of any novel tool or organizational process, a number of directions for future research warrant consideration. First, because the PHO Risk Screening Tool was developed and validated in a Canadian public health setting, additional validation in diverse settings is recommended to verify the external validity of the Risk Screening Tool in other settings and regions. Moreover, given that the tool was developed in the absence of a gold standard for scoring or appraising the level of risk associated with diverse health-related evidence-generating initiatives, we invite feedback on the scores assigned to each fixed response in the PHO Risk Screening Tool.

## Conclusion

Efforts to facilitate a paradigmatic shift from the prevailing distinction between research and non-research endeavors are underway [6]. A key factor limiting the implementation of this shift in ideology is the question of how to ensure appropriate risk management and ethical oversight of evidence-generating initiatives. The PHO Risk Screening Tool offers a practical solution to the problem of how to maintain accountability and appropriate risk oversight that transcends the boundaries of research and practice. We hope that the PHO Risk Screening Tool will prove useful in minimizing the problems of over and under protection across a wide range of disciplines and jurisdictions.

### Availability and requirements

**Project name:** Public Health Ontario Risk Screening Tool

**Project home page:**http://www.publichealthontario.ca/en/ServicesAndTools/ResearchAndEducationSupport/Pages/Ethics-support-forms-and-tools.aspx

**Operating system:** Platform independent

**Programming language:** Javascript

**Other requirments:** JavaScript enabled browser, IE8+ or any modern browser (Chrome, Firefox, Safari)

**License:** None

**Any restrictions to use by non-academics:** Non-commercial use only. Please credit Public Health Ontario. No modification without permission.

## Additional file

Additional file 1:
**Public Health Ontario Risk Screening Tool 2.0.**

## Abbreviations

ERB: Ethics Review Board
PHO: Public Health Ontario
